# Nurse–Patient Mutuality and Health-Related Quality of Life Among Nurses Caring for People with Chronic Illness: A Multicentre Cross-Sectional Study

**DOI:** 10.3390/nursrep16090300

**Published:** 2026-08-25

**Authors:** Camilla Ripari, Silvia Cilluffo, Barbara Bassola, Rosario Caruso, Stefano Terzoni, Maura Lusignani

**Affiliations:** 1 Department of Biomedicine and Prevention, University of Rome “Tor Vergata”, 00133 Rome, Italy; camilla.ripari@students.uniroma2.eu; 2 School of Nursing, ASST Grande Ospedale Metropolitano Niguarda, 20162 Milan, Italy; barbara.bassola@ospedaleniguarda.it (B.B.); maura.lusignani@unimi.it (M.L.); 3 Department of Biomedical Sciences for Health, University of Milan, 20133 Milan, Italy; rosario.caruso@unimi.it (R.C.); stefano.terzoni@unimi.it (S.T.); 4 Health Professions Research Unit and Evidence Transfer, IRCCS MultiMedica, 20099 Sesto San Giovanni, Italy

**Keywords:** mutuality, nurse–patient relations, quality of life, nurses, linear models

## Abstract

**Background**: Although nurses’ health-related quality of life (HRQoL) is critical for mitigating the growing intention to leave the profession, the association between nurse–patient mutuality and nurses’ HRQoL remains unexplored. Therefore, this study aims to examine the association between nurse–patient mutuality and nurses’ HRQoL. **Methods**: A cross-sectional, multicentre study was conducted across four tertiary hospitals in Italy. Data were collected from a sample of 726 nurses working in medical and surgical inpatient and outpatient wards. Mutuality was measured using the Nurse–patient Mutuality in Chronic Illness scale, comprising three dimensions: developing and going beyond, being a point of reference, deciding and sharing care; HRQoL was assessed via the Short Form-12 Health Status Questionnaire (SF-12), providing Physical (PCS) and Mental (MCS) Component Summaries. Two multiple linear regression models were conducted to evaluate the impact of mutuality dimensions on HRQoL components. **Results**: Among the 726 participants, none of the three mutuality dimensions were significantly associated with physical HRQoL (PCS). Conversely, two mutuality dimensions were significantly and positively associated with higher MCS scores: developing and going beyond (B = 0.54, 95% CI [0.14, 0.95], *p* = 0.009) and being a point of reference (B = 0.38, 95% CI [0.09, 0.68], *p* = 0.010). However, the overall regression model demonstrated a modest explanatory value for MCS (R^2^ = 0.075). Among covariates, female sex (ref: male; B = −2.29, 95% CI [−4.10, −0.49], *p* = 0.013) and taking care of relatives (B = −2.07, 95% CI [−3.55, −0.60], *p* = 0.006) were associated with lower MCS, whereas older age (B = 0.17, 95% CI [0.01, 0.34], *p* = 0.039) was related to higher MCS. Regarding physical health (R^2^ = 0.261), having chronic conditions (B = −5.08, 95% CI [−6.48, −3.69], *p* < 0.001), previous experience as a patient (B = −1.88, 95% CI [−3.07, −0.69], *p* = 0.002), and taking care of relatives (B = −1.49, 95% CI [−2.62, −0.36], *p* = 0.010) were significantly associated with lower PCS scores. **Conclusions**: The reciprocal nature of mutuality could be a potential relational resource for nurses’ mental HRQoL. Healthcare organizations should prioritize structural frameworks and interventions that allow nurses the time and resources to cultivate these essential clinical connections.

## 1. Introduction

Nurses play an indispensable role in healthcare delivery, encompassing compassionate, person-centred care and service management [1]. In the context of persistent global workforce pressures and high job strain [2], supporting nurses’ health-related quality of life (HRQoL) has become a crucial priority for sustaining a healthy healthcare workforce [3]. In contrast to general quality of life—which encompasses overall life satisfaction, culture, and personal goals—HRQoL is a distinct, multidimensional construct specifically capturing the impact of health status on an individual’s physical, mental, and social functioning [4]. In empirical research, HRQoL is commonly evaluated through standardized generic health surveys such as the SF-12, yielding two primary summary scores: the Physical Component Summary (PCS) and the Mental Component Summary (MCS) [5]. While previous research indicates that nurses’ HRQoL is shaped by stress, health behaviours, personal and job-related characteristics, and the perceived work environment [6], the specific contribution of nurse–patient relationships remains underexplored. Extant studies predominantly address interpersonal relationships, in a broad sense, conflating interactions with peers, physicians, patients, and family members [6].

Several theoretical and empirical perspectives suggest that high-quality therapeutic relationships may represent an important psychosocial resource for nurses. Nursing practice requires substantial emotional labour, involving the continuous regulation of emotions while responding to patients’ needs and maintaining compassionate care. Evidence indicates that emotional labour, work-related stress, and role-related demands are closely associated with nurses’ psychological and occupational well-being [7]. Conversely, supportive relational experiences may foster emotional regulation, professional fulfilment, and adaptive coping strategies in clinical practice [8]. Positive nurse–patient relationships have been associated with favourable occupational outcomes, including higher job satisfaction and professional well-being, supporting the view that relational resources may contribute to clinicians’ overall health and functioning [9]. Furthermore, relational nursing theories emphasize that therapeutic nurse–patient relationships are built through empathy, mutual understanding, emotional connection, and meaningful interpersonal interactions, which may help nurses cope with the emotional demands of clinical practice and reinforce professional fulfilment and role meaning [10,11].

However, the nurse–patient relationship is increasingly recognized as a vital determinant of nurses’ well-being, particularly when it is characterized by mutuality, namely, trust, reciprocity, empathy, and respect [12]. Nurse–patient mutuality is a dynamic process structured around three key dimensions: developing and going beyond, being a point of reference, and deciding and sharing care [12,13]. High mutuality yields positive outcomes for both nurses and patients [12]. Among nurses, greater mutuality has been linked to enhanced professional quality of life, particularly through higher compassion satisfaction and reduced work-related fatigue [14]. For patients, positive nurse–patient relationships foster better quality of life [15], and nurse–patient mutuality in the chronic-care context has been shown to influence HRQoL for both parties [16]. Because mutuality represents a relational and psychosocial resource rooted in emotional connection, shared decision-making, and psychological safety, its primary influence is expected to operate on mental health mechanisms (MCS) rather than physical functioning (PCS). However, while initial dyadic research suggests a positive link between overall mutuality and nurses’ mental health [16], empirical research directly exploring how specific dimensions of mutuality relate to nurses’ HRQoL in a large, independent sample of nurses remains limited. Examining these specific dimensions is essential to identify which relational aspects most strongly support nurse HRQoL. To address this gap, the present study aims to examine the association between specific dimensions of nurse–patient mutuality and nurses’ HRQoL in a multicentre sample of *N* = 726 nurses. Given the limited prior literature on dimension-specific effects, analyses evaluating the three individual mutuality dimensions in relation to HRQoL were considered exploratory.

## 2. Materials and Methods

### 2.1. Design

A cross-sectional, multicentre study conducted across four Italian tertiary hospitals was designed to explore the relationship between nurse–patient mutuality and nurses’ HRQoL. This observational study was conducted and reported in accordance with the Strengthening the Reporting of Observational Studies in Epidemiology (STROBE) statement (Supplementary Materials Checklist S1).

### 2.2. Setting and Sample

The study was conducted in four tertiary hospitals in Italy. Data were collected between January 2025 and December 2025. A convenience sampling strategy was used to enrol nurses working in chronic illness inpatient and outpatient settings. The study population consisted of nurses who cared for patients with chronic illness.

To calculate the required sample size, an a priori power calculation was performed using G*Power (version 3.1.9.7) for multiple linear regression models (F tests, linear multiple regression: fixed model, R^2^ increase) [17]. Based on previous research on nurse-related psychosocial correlates of HRQoL reporting small-to-moderate associations (f^2^ = 0.02 to 0.05) [16], and in the absence of prior dimension-specific mutuality data, we conservatively assumed a small effect size of f^2^= 0.02. Assuming an α level of α = 0.05, a target power (1 − β) of 0.80, three focal independent variables (the three NPM-CI subscales), and a total of 12 variables (including demographic and clinical covariates), the minimum required sample size was 550 participants for overall R^2^ evaluation (and 550 for testing the incremental R^2^ increase in the three focal mutuality dimensions). Accounting for an anticipated 20% non-response or incomplete rate, the target recruitment was set to at least 660 nurses. The final analytical sample of *N* = 726 complete cases exceeded this threshold, ensuring adequate statistical power.

### 2.3. Inclusion and Exclusion Criteria

The inclusion criteria were at least one year of nursing work experience, working with patients with chronic illness in inpatient or outpatient settings, willingness to participate, and provision of written informed consent. These criteria were established to ensure that all participants had sufficient clinical experience with chronic illness care to meaningfully evaluate nurse–patient mutuality, while focusing on settings in which mutuality is most likely to develop. No additional exclusion criteria were applied beyond not meeting the specified inclusion criteria.

### 2.4. Instruments

Nurses’ HRQoL was measured using the validated Italian version of the 12-Item Short Form Health Survey (SF-12v1) [5,18]. The instrument provides two standardized summary scores, namely the Physical Component Summary (PCS) and the Mental Component Summary (MCS), calculated using standard scoring algorithms [5,18]. PCS and MCS are reported as norm-based scores, linearly transformed to a population mean of 50 and a standard deviation of 10, where higher scores indicate better health status. In the Italian validation study, internal consistency was demonstrated with Cronbach’s α values of 0.85 for the PCS and 0.77 for the MCS [18]. Permission and licencing for survey administration were obtained prior to data collection.

Mutuality was measured using the nurse version of the Nurse–patient Mutuality in Chronic Illness (NPM-CI) scale, a self-assessment tool comprising 20 items rated on a five-point Likert scale from 1 (never) to 5 (always) [13]. The scale evaluates three specific dimensions: developing and going beyond (four items, raw score range: 4–20), being a point of reference (seven items, raw score range: 7–35), and deciding and sharing care (nine items, raw score range: 9–45). Higher total scores reflect greater nurse–patient mutuality. Missing item responses were handled via listwise deletion, including only complete cases in the analyses. While the original nurse version demonstrated high overall internal consistency (Cronbach’s α = 0.90) and test–retest reliability (ICC = 0.96, 95% CI 0.89–0.99; *p* < 0.001) [13], internal consistency in the present sample (*N* = 726) was good across all subscales: developing and going beyond (α = 0.748, ω = 0.759), being a point of reference (α = 0.774, ω = 0.781), and deciding and sharing care (α = 0.798, ω = 0.801).

Age, sex, years of professional experience, education, postgraduate education, work setting, previous experience as a patient, taking care of relatives, and having chronic conditions were used as control variables. These covariates were selected in line with the conceptual framework [12]. Previous research indicates that increased age is associated with worse physical health but better mental health, and more years of work experience are associated with better mental health [6]. Higher levels of education have been associated with a lower workload and better HRQoL [19]. Male sex has shown an association with higher HRQoL [20], whereas having chronic conditions and taking care of relatives were associated with lower HRQoL [21]. Working in outpatient care settings has been linked to superior HRQoL and work–life balance [22], and having previous experience as a patient may influence nurses’ HRQoL in either a positive or negative manner [23].

### 2.5. Data Collection

Research staff received on-site training to facilitate data collection among nurses across four tertiary hospitals in Northern and Central Italy. Members of the research team visited the clinical departments directly to approach potentially eligible nurses and administer the study protocol in person. Data collection was conducted during nurses’ shift breaks to facilitate participation without interfering with clinical activities. Participation was entirely voluntary, and nurse managers were not involved in questionnaire administration or collection to prevent potential coercion. Each participating nurse contributed only one questionnaire. Participant recruitment continued until the target sample size was achieved. Overall, 800 nurses were approached and assessed for eligibility; 50 nurses were excluded (35 did not meet the inclusion criteria and 15 declined to participate), resulting in 750 enrolled participants. Prior to enrolment, sampling criteria were thoroughly evaluated, and all participants provided written informed consent. Data collectors were trained to administer the study protocol standardly and assist with any procedural queries. The questionnaires were self-administered and took approximately 15–20 min to complete. To protect participant privacy and maintain voluntary participation while data collectors remained in the room to address queries, questionnaires were completed individually, placed into blank sealed envelopes upon completion, and deposited into a locked collection box. To safeguard participant confidentiality, centre-level identifiers and specific operational unit names were not recorded in the consolidated dataset; instead, clinical environments were categorized into inpatient and outpatient settings, alongside participants’ overall employee tenure. Of the 750 enrolled participants, 726 fully completed the questionnaire (completion rate: 96.8%). The remaining 24 questionnaires were incomplete due to omitted items across demographic or clinical sections and were excluded, yielding a final complete-case analytical sample of *N* = 726.

### 2.6. Data Analysis

Descriptive statistics were used to calculate means, standard deviations, and percentages for categorical variables, as appropriate, along with linear correlations between the mutuality dimensions and the HRQoL subscales. Missing data were evaluated at both the item and individual levels. Because missingness was minimal (<2% across all variables), advanced imputation techniques were deemed unnecessary, and a complete-case analysis approach was applied. Consequently, only participants with complete data across all study variables were included in the descriptive statistics and regression models.

To address the research question, multiple linear regression analyses were conducted. Specifically, the three dimensions of mutuality were entered simultaneously into each model and regressed against PCS and MCS scores. All regressions were adjusted for predefined covariates: age, sex, years of work experience, education, postgraduate education, work setting (inpatient/outpatient), previous experience as a patient, caregiving for relatives, and presence of chronic conditions. Dichotomous variables were dummy-coded prior to entry into the models (e.g., Sex: 0 = male [reference category] 1 = female; Work setting: 0 = inpatient [reference category], 1 = outpatient; Taking care of relatives: 0 = no, 1 = yes; Previous experience as a patient: 0 = no, 1 = yes; Having chronic conditions: 0 = no, 1 = yes). Regression assumptions were thoroughly evaluated: linearity, homoscedasticity, and normality of model residuals were assessed graphically using residual-versus-fitted plots and Q-Q plots. While the Shapiro–Wilk test was formally calculated, evaluation relied primarily on graphical residual diagnostics given the known over-sensitivity of formal normality tests in large sample sizes (*N* = 726). Residual independence was confirmed via the Durbin–Watson test (DW = 1.85). To quantify the incremental variance contributed by mutuality beyond covariates, two-block hierarchical linear regressions were performed, reporting ΔR^2^ and Cohen’s f^2^ effect sizes.

Multicollinearity was evaluated using Variance Inflation Factors (VIFs) and Tolerance statistics: all mutuality dimensions showed low values (VIF = 1.55–2.05; Tolerance > 0.488; threshold VIF < 5), while age (VIF = 6.54; Tolerance = 0.153) and work experience (VIF = 7.50; Tolerance = 0.133) showed moderate multicollinearity, reflecting the expected conceptual and empirical overlap between these variables. Both variables were retained because age and professional experience represent distinct demographic and occupational characteristics that may independently contribute to nurses’ quality of life. To assess the robustness of the findings, sensitivity analyses were performed by repeating the regression models after separately removing age and work experience. The estimates for the three mutuality dimensions remained substantially unchanged, supporting the stability of the results. Finally, influential observations were ruled out using Cook’s distance (maximum = 0.0253, threshold < 1.0).

To account for multiple testing across the family of six primary focal coefficients (evaluating the three NPM-CI dimensions across both MCS and PCS regression models), *p*-values were adjusted using the Benjamini–Hochberg False Discovery Rate (FDR) procedure. Both unadjusted (p_unadjusted_) and FDR-adjusted (p_adj_) values are reported. The statistical significance level for all analyses was set at *p* < 0.05; all calculations were performed using Jamovi (version 2.5 for Windows) [24].

### 2.7. Ethical Considerations

All nurses provided written informed consent prior to enrolment, and the study was conducted in accordance with the principles of the Declaration of Helsinki. Ethical approval was granted by the Ethics Committee of CEMIA3 (Comitato Etico Milano Area 3), located in Milan, on 22 July 2021 (registration number 501-22072021). This approval, along with local hospital authorizations, covered data collection across all four participating hospitals during January 2025–December 2025. The study was not registered.

## 3. Results

### 3.1. Sample Characteristics

Seven hundred and fifty nurses were recruited, of whom seven hundred and twenty-six completed the study (96.8%). The remaining 24 incomplete questionnaires were excluded, yielding a final complete-case analytical sample of *N* = 726 with no missing values. Of these, five hundred and sixty-eight (78.2%) were women. The mean age was 39.4 years (SD = 11.1), with a mean of 14.8 years (SD = 11.2) of nursing working experience. Overall, four hundred and sixty-nine (64.6%) held a bachelor’s degree in nursing, and five hundred and eighteen (71.3%) had not completed any postgraduate education. Five hundred and six (69.7%) were nurses in an inpatient setting. Three hundred and eighty-two (52.6%) had previous experience as a patient, three hundred and sixty-seven (50.6%) were taking care of relatives, and five hundred and fifty-seven (76.7%) had no chronic conditions. Detailed participant characteristics are presented in Table 1.

### 3.2. Descriptive Analysis and Correlations

The descriptive analysis for the Pearson linear correlation coefficient r between the mutuality and HRQoL dimensions is shown in Table 2.

### 3.3. Multivariate Linear Regression

A multiple linear regression was conducted to examine whether the three dimensions of mutuality—developing and going beyond, being a point of reference and deciding and sharing care—were associated with MCS. The overall model explained 7.5% of the variance in MCS. Both the developing and going beyond dimension (β = 0.118, B = 0.54 [0.137, 0.949], t = 2.62, *p* = 0.009) and the being a point of reference dimension (β = 0.122, B = 0.38 [0.093, 0.676], t = 2.59, *p* = 0.010) was significantly and positively associated with MCS. Among the covariates, female sex (coded as one vs. zero = male; β = −0.094, B = −2.293 [−4.096, −0.491], t = −2.50, *p* = 0.013) and taking care of relatives (β = −0.103, B = −2.07 [−3.546, −0.595], t = −2.76, *p* = 0.006) were significantly and negatively associated with MCS, and that higher age was significantly associated with MCS (β = 0.191, B = 0.17 [0.009, 0.339], t = 2.07, *p* = 0.039). Complete regression results for MCS are presented in Table 3.

To test whether the three dimensions of mutuality were associated with PCS, a second multiple linear regression was evaluated, explaining 26.1% of the variance in PCS. None of the three dimensions of mutuality were significantly associated with PCS. Among the covariates, having chronic conditions (β = −0.249, B = −5.08 [−6.477, −3.686], t = −7.16, *p* < 0.001), previous experience as a patient (β = −0.109, B = −1.88 [−3.065, −0.694], t = −3.11, *p* = 0.002), and taking care of relatives were significantly and negatively associated with PCS (β = −0.086, B = −1.49 [−2.617, −0.360], t = −2.59, *p* = 0.010).

In hierarchical models, adding the mutuality block yielded a non-significant variance increase for PCS (ΔR^2^ = 0.0038, F(3, 705) = 1.24, *p* = 0.293, f^2^ = 0.004) and a statistically significant, though small, increase for MCS (ΔR^2^ = 0.0225, F(3, 708) = 5.78, *p* < 0.001, f^2^ = 0.023).

In the MCS model, two dimensions retained statistical significance after Benjamini–Hochberg FDR adjustment (p_adj_ = 0.030 for both), whereas deciding and sharing care remained non-significant (p_adj_ = 0.269). In the PCS model, none of the three NPM-CI dimensions reached statistical significance before or after multiple-testing correction (p_adj_ > 0.264 for all dimensions).

Sensitivity analyses re-estimating the regression models after separately omitting age or work experience confirmed the stability of the estimates: coefficients for all NPM-CI dimensions showed negligible variations (ΔB < 0.01), and overall model fit remained virtually unchanged for both PCS (R^2^ = 0.255–0.261) and MCS (R^2^ = 0.071–0.075).

Complete regression results for PCS are presented in Table 4.

## 4. Discussion

Our findings replicate and refine the earlier evidence reported by our research group [16], which identified an association between overall nurse–patient mutuality and nurses’ HRQoL measured via the SF-12. By extending our previous work to the specific dimension level, our results suggest that two components (developing and going beyond, being a point of reference) could be associated with the mental component of nurses’ HRQoL. The dimension developing and going beyond is defined by empathy and a holistic approach that recognizes the patient as a whole person. These findings align with the existing literature, demonstrating that empathy and emotional intelligence serve as significant correlates of nurses’ mental and psychological well-being [25]. Similarly, the dimension being a point of reference is characterized by the pivotal role of nurses in patient care and their ability to ensure continuity of care. Beyond its established association with job satisfaction [14], our findings suggest that this dimension may be associated with nurses’ mental quality of life. Conversely, the shift in deciding and sharing care from a positive bivariate correlation to a non-significant multivariable coefficient could reflect shared variance among the NPM-CI subscales, suggesting that its bivariate contribution is largely accounted for by the deeper empathetic dimensions. However, it is important to note that the regression model predicting MCS explains only 7.5% of the overall variance (R^2^ = 0.075). While these associations are statistically significant, the empirical contribution of nurse–patient mutuality remains modest, and the clinical weight of these findings should be further investigated with future longitudinal studies to confirm these preliminary results.

Regarding the covariates, our findings are consistent with the broader literature. Specifically, taking care of relatives was significantly associated with lower scores in both PCS and MCS dimensions, consistent with prior research [21]. Indeed, the responsibilities inherent in being a family caregiver are often linked to diminished mental and physical HRQoL, particularly among healthcare professionals who must balance these informal duties with the demanding requirements of a nursing role [21]. Female sex was significantly associated with lower MCS scores relative to the male reference category (0 = male, 1 = female), reflecting higher overall mental HRQoL among male nurses, in line with Babapour et al. [20]. This disparity aligns with broader epidemiological trends in health-related quality of life reporting. Moreover, older age was significantly associated with higher MCS scores, echoing the findings of Oyama et al. [6].

Furthermore, the presence of chronic conditions was significantly associated with lower PCS scores. This confirms prior studies highlighting the negative impact of chronic illness on the physical HRQoL among healthcare professionals [21]. Prior personal experience as a patient was likewise associated with lower PCS scores. While research on the impact of such experiences on nurses’ physical HRQoL remains scarce [23,26], this association should be interpreted with caution, as it may reflect residual confounding by underlying health history, disease severity, or chronic subclinical conditions that prompt healthcare utilization rather than a direct mechanism.

These associations can be interpreted through established theoretical perspectives on relational work and clinician well-being [14]. Mutuality may enhance nurses’ mental HRQoL by functioning as a relational resource that promotes emotional regulation, shared meaning-making, and perceived reciprocity within the therapeutic relationship. According to relational nursing theories [10,11], high-quality interpersonal exchanges help nurses experience greater role clarity, emotional connection, and professional efficacy, which in turn buffer the psychological effects of work stress and emotional labour [10,11]. Mutual relationships may therefore foster a sense of coherence and purpose, reinforcing nurses’ intrinsic motivation and contributing to improved mental well-being [27]. This theoretical mechanism may explain why the dimensions developing and going beyond and being a point of reference are particularly influential for mental HRQoL, as they reflect deeper empathic engagement and a stable relational presence in care.

### 4.1. Implications for Policy and Practice

Current healthcare policies increasingly emphasize patient-centred care models; however, systemic challenges, such as chronic nursing shortages and escalating workloads, frequently complicate their practical application [28]. Notably, nursing shortages show complex associations with nurses’ HRQoL, which is hypothesized to co-vary with the implementation of relational care models [29]. Our findings indicate that nurse–patient mutuality is positively associated with nurses’ mental HRQoL. In clinical environments, relational models rooted in reciprocity and empathy—transcending traditional professional boundaries and enabling nurses to act as a consistent point of reference for patients—correlate with higher mental well-being among nursing staff. Consequently, future prospective and intervention-based studies are needed to evaluate whether healthcare organizational models prioritizing patient-centred care and adequate dedicated time are linked to improved, long-term nurse-reported outcomes.

### 4.2. Recommendations for Further Research

As this study represents an initial exploration into how mutuality is associated with nurses’ HRQoL, it provides a foundation for several lines of future inquiry. Future research should prioritize longitudinal designs to establish causality and elucidate the trajectory of nurses’ mental HRQoL over time. To enhance external validity, international and cross-cultural studies are essential to validate these preliminary findings across diverse healthcare systems and nursing traditions. Moreover, given that developing and going beyond and being a point of reference emerged as pivotal dimensions, future investigations should employ qualitative or mixed-method approaches to investigate how these specific relational competencies are cultivated in daily clinical practice. Such research will be invaluable for refining nursing education and informing targeted interventions designed to improve staff retention and professional satisfaction.

### 4.3. Strengths and Limitations

This study possesses several noteworthy strengths. To our knowledge, it represents the first empirical investigation into the specific association between nurse–patient mutuality and nurses’ HRQoL, addressing a significant gap in the literature regarding relational competencies and healthcare providers well-being. Furthermore, the recruitment of a robust sample from multiple hospitals and diverse clinical settings improves statistical precision and sample heterogeneity. By identifying that two specific mutuality dimensions—developing and going beyond and being a point of reference—were associated with nurses’ mental HRQoL, this research evaluates these relationships within an established relational framework, offering insights for future research aimed at supporting nursing staff well-being.

Despite these contributions, several limitations must be acknowledged. First, the cross-sectional design precludes inferring definitive causal relationships and cannot rule out reverse causality. Second, while the sample size is substantial, the reliance on a convenience sample from Italian tertiary chronic-care settings constrains generalizability to other healthcare systems. Third, unmeasured organizational and occupational variables—such as specific leadership styles, unit culture, staffing, shift patterns or burnout—may represent residual confounding with mental HRQoL. Fourth, because hospital and department identifiers were omitted from the database to preserve full participant anonymity, it was not possible to fit clustered or fixed-effects models; this unobserved nesting represents a methodological limitation that may have affected standard errors and confidence intervals. Fifth, the use of self-reported measures, although standard in HRQoL assessment, remains susceptible to common-method variance, as well as social desirability and recall biases. Lastly, the full MCS regression model explained a modest proportion of variance (R^2^ = 0.075), with small standardized coefficients (β = 0.118 and 0.122) and a small incremental variance accounted for by mutuality (ΔR^2^ = 0.0225, f^2^ = 0.023). Thus, its direct clinical relevance should be interpreted cautiously as one modest factor within a complex network of organizational and personal determinants, and these exploratory findings warrant confirmation in future prospective studies.

## 5. Conclusions

This study provides empirical evidence on how specific dimensions of nurse–patient mutuality relate to nurses’ HRQoL. Specifically, the mutuality dimensions of developing and going beyond and being a point of reference emerged as correlates of nurses’ mental HRQoL. These results suggest that mutuality may represent a relational resource that could support the psychological well-being of the nursing workforce. While individual and clinical factors—such as caregiving duties (associated with lower PCS and MCS) and chronic conditions (associated with lower PCS)—were associated with poorer HRQoL, the reciprocal nature of the nurse–patient relationship appears to be positively associated with nurses’ mental health. Future prospective research should evaluate whether healthcare organizations could benefit from structural conditions that afford nurses the time and environment necessary to cultivate meaningful therapeutic relationships. Strengthening these relational foundations should be explored as a potential strategy to support nurse well-being.

## Figures and Tables

**Table 1:** Demographic and clinical characteristics of the sample.

Variable	Total Sample (*N* = 726)
Age mean (sd)	39.4 (11.1)
Sex	
Male	158 (21.8%)
Female	568 (78.2%)
Working years as a nurse mean (sd)	14.8 (11.2)
Undergraduate education	
Diploma in Nursing—pre-BScN ^1^	199 (27.4%)
BScN ^1^	469 (64.6%)
Professional Diploma in Nursing	58 (8.0%)
Postgraduate education	
None	518 (71.3%)
Clinical postgraduate diploma	127 (17.5%)
Postgraduate diploma in management	55 (7.6%)
MScN ^2^	26 (3.6%)
Employee tenure	
Inpatient setting	506 (69.7%)
Outpatient setting	220 (30.3%)
Previous experience as patient	
No	344 (47.4%)
Yes	382 (52.6%)
Taking care of relatives	
No	359 (49.4%)
Yes	367 (50.6%)
Having chronic conditions	
No	557 (76.7%)
Yes	169 (23.3%)

^1^ BScN Bachelor of Science in Nursing, ^2^ MScN Master of Science in Nursing.

**Table 2:** Descriptive statistics and correlation of mutuality dimensions and HRQoL dimensions.

Dimensions	Mean (SD)	DGB	PR	DSC	PCS	MCS
DGB ^1^	17.3 (2.2)	-				
PR ^2^	27.0 (3.2)	0.426 **	-			
DSC ^3^	35.0 (4.7)	0.578 **	0.625 **	-		
PCS ^4^	50.6 (8.6)	0.007	−0.048	−0.028	-	
MCS ^5^	46.5 (10.1)	0.135 **	0.158 **	0.099 *	−0.131 **	-

^1^ DGB = developing and going beyond; ^2^ PR = being a point of reference; ^3^ DSC = deciding and sharing care; ^4^ PCS = Physical Component Summary; ^5^ MCS = Mental Component Summary. * *p* < 0.05, ** *p* < 0.001. Note: NPM-CI subscale scores are expressed as raw total scores: developing and going beyond (4 items, range 4–20), being a point of reference (7 items, range 7–35), and deciding and sharing care (9 items, range 9–45); higher scores indicate higher mutuality. PCS and MCS represent norm-based T-scores (mean = 50, SD = 10; theoretical range: 0–100, where higher scores reflect better physical/mental HRQoL).

**Table 3:** Multiple linear regression model of nurse–patient mutuality on MCS with the covariates.

Variable	β	B [95%CI]	t	*p*	R^2^
					0.075
Developing and going beyond	0.118	0.549 [0.137; 0.949]	2.624	0.009	
Being a point of reference	0.122	0.385 [0.093; 0.676]	2.589	0.010	
Deciding and sharing care	−0.069	−0.149 [−0.366; 0.068]	−1.344	0.179	
Age	0.191	0.174 [0.009; 0.339]	2.068	0.039	
Sex (female)	−0.094	−2.293 [−4.096; −0.491]	−2.498	0.013	
Working years	−0.039	−0.035 [−0.209; 0.140]	−0.390	0.697	
Education (undergraduate)	−0.027	−0.474 [−2.045; 1.096]	−0.593	0.553	
Education (postgraduate)	−0.006	−0.071 [−1.030; 0.889]	−0.145	0.885	
Employee tenure	−0.002	−0.042 [−1.965; 1.881]	−0.043	0.966	
Previous experience as patient	−0.026	−0.512 [−2.062; 1.039]	−0.648	0.517	
Taking care of relatives	−0.103	−2.070 [−3.546; −0.595]	−2.755	0.006	
Having chronic conditions	−0.024	−0.577 [−2.403; 1.248]	−0.621	0.535	

Note: *N* = 726. Model F(12, 713) = 4.80, *p* < 0.001, R^2^ = 0.075, Adjusted R^2^ = 0.059. B = unstandardized regression coefficient; β = standardized regression coefficient; CI = confidence interval; Reference categories for categorical variables are described in the Methods section. NPM-CI dimensions are evaluated as raw subscale total scores. Adjusted *p*-values reflect Benjamini–Hochberg False Discovery Rate (FDR) correction applied across the family of six focal NPM-CI subscale coefficients (MCS and PCS models combined). p_adj_ values for the three NPM-CI dimensions are 0.030 (developing and going beyond), 0.030 (being a point of reference), and 0.269 (deciding and sharing care). Note for hierarchical Block 2 (mutuality added): ΔR^2^ = 0.0225, F(3, 708) = 5.78, *p* < 0.001, f^2^ = 0.023.

**Table 4:** Multiple linear regression model of nurse–patient mutuality on PCS with the covariates.

Variable	β	B [95%CI]	t	*p*	R^2^
					0.261
Developing and going beyond	0.060	0.235 [−0.075; 0.546]	1.487	0.138	
Being a point of reference	−0.032	−0.086 [−0.310; 0.137]	−0.761	0.447	
Deciding and sharing care	0.036	0.067 [−0.099; 0.233]	0.788	0.431	
Age	−0.129	−0.100 [−0.227; 0.026]	−1.564	0.118	
Sex (female)	0.014	0.299 [−1.080; 1.677]	0.425	0.671	
Working years	−0.152	−0.117 [−0.250; 0.017]	−1.718	0.086	
Education (undergraduate)	0.024	0.368 [−0.833; 1.569]	0.602	0.547	
Education (postgraduate)	0.013	0.146 [−0.588; 0.879]	0.389	0.697	
Employee tenure	−0.025	−0.473 [−1.943; 0.998]	−0.631	0.528	
Previous experience as patient	−0.109	−1.880 [−3.065; −0.694]	−3.113	0.002	
Taking care of relatives	−0.086	−1.488 [−2.617; −0.360]	−2.590	0.010	
Having chronic conditions	−0.249	−5.081 [−6.477; −3.686]	−7.157	<0.001	

Note: *N* = 726. Model F(12, 713) = 21.0, *p* < 0.001, R^2^ = 0.261, Adjusted R^2^ = 0.248. B = unstandardized regression coefficient; β = standardized regression coefficient; CI = confidence interval; Reference categories for categorical variables are described in the Methods section. NPM-CI dimensions are evaluated as raw subscale total scores. Adjusted *p*-values reflect Benjamini–Hochberg False Discovery Rate (FDR) correction applied across the family of six focal NPM-CI subscale coefficients (MCS and PCS models combined). p_adj_ values for the three NPM-CI dimensions are 0.264 (developing and going beyond), 0.447 (being a point of reference), and 0.447 (deciding and sharing care). Note for hierarchical Block 2 (mutuality added): ΔR^2^ = 0.0038, F(3, 705) = 1.24, *p* = 0.293, f^2^ = 0.004.

## Data Availability

The raw data supporting the conclusions of this article will be made available by the authors on request.

## Supplementary Materials

The following supporting information can be downloaded at: https://www.mdpi.com/article/10.3390/nursrep16090300/s1, Checklist S1: STROBE Statement—Checklist of items that should be included in reports of cross-sectional studies [30].

## Public Involvement Statement

No public involvement in any aspect of this research.

## Guidelines and Standards Statement

This manuscript was drafted against the Strengthening the Reporting of Observational Studies in Epidemiology (STROBE) for observational studies.

## Abbreviations

The following abbreviations are used in this manuscript:

HRQoL	Health-Related Quality of Life
SF-12	Short Form-12 Health Status Questionnaire
PCS	Physical Component Summary
MCS	Mental Component Summary
NPM-CI	Nurse–patient Mutuality in Chronic Illness scale
ICC	Intraclass Correlation Coefficient
SD	Standard Deviation
BScN	Bachelor of Science in Nursing
MScN	Master of Science in Nursing
DGB	Developing and Going Beyond
PR	Being a Point of Reference
DSC	Deciding and Sharing Care
